# Genomic Determinants of Triglyceride and Cholesterol Distribution into Lipoprotein Fractions in the Rat

**DOI:** 10.1371/journal.pone.0109983

**Published:** 2014-10-08

**Authors:** Miloslava Hodúlová, Lucie Šedová, Drahomíra Křenová, František Liška, Michaela Krupková, Ludmila Kazdová, Johanne Tremblay, Pavel Hamet, Vladimír Křen, Ondřej Šeda

**Affiliations:** 1 Institute of Biology and Medical Genetics, the First Faculty of Medicine, Charles University and the General Teaching Hospital, Prague, Czech Republic; 2 Department of Metabolism and Diabetes, Institute for Clinical and Experimental Medicine, Prague, Czech Republic; 3 Centre de recherche, Centre hospitalier de l’Université de Montréal (CRCHUM) – Technôpole Angus, Montreal, Quebec, Canada; 4 Institute of Molecular Genetics, Academy of Sciences of the Czech Republic, Prague, Czech Republic; University of Colorado Denver, United States of America

## Abstract

The plasma profile of major lipoprotein classes and its subdivision into particular fractions plays a crucial role in the pathogenesis of atherosclerosis and is a major predictor of coronary artery disease. Our aim was to identify genomic determinants of triglyceride and cholesterol distribution into lipoprotein fractions and lipoprotein particle sizes in the recombinant inbred rat set PXO, in which alleles of two rat models of the metabolic syndrome (SHR and PD inbred strains) segregate together with those from Brown Norway rat strain. Adult male rats of 15 PXO strains (n = 8–13/strain) and two progenitor strains SHR-*Lx* (n = 13) and BXH2/Cub (n = 18) were subjected to one-week of high-sucrose diet feeding. We performed association analyses of triglyceride (TG) and cholesterol (C) concentrations in 20 lipoprotein fractions and the size of major classes of lipoprotein particles utilizing 704 polymorphic microsatellite markers, the genome-wide significance was validated by 2,000 permutations per trait. Subsequent *in silico* focusing of the identified quantitative trait loci was completed using a map of over 20,000 single nucleotide polymorphisms. In most of the phenotypes we identified substantial gradient among the strains (e.g. VLDL-TG from 5.6 to 66.7 mg/dl). We have identified 14 loci (encompassing 1 to 65 genes) on rat chromosomes 3, 4, 7, 8, 11 and 12 showing suggestive or significant association to one or more of the studied traits. PXO strains carrying the SHR allele displayed significantly higher values of the linked traits except for LDL-TG and adiposity index. Cholesterol concentrations in large, medium and very small LDL particles were significantly associated to a haplotype block spanning part of a single gene, low density lipoprotein receptor-related protein 1B (*Lrp1b*). Using genome-wide association we have identified new genetic determinants of triglyceride and cholesterol distribution into lipoprotein fractions in the recombinant inbred panel of rat model strains.

## Introduction

Utilization of total concentrations of cholesterol (C) and triacylglycerols (TG) together with cholesterol levels in high and low density lipoprotein particles (HDL and LDL, respectively) has been a mainstay of cardiovascular risk assessment [1]. However, recent evidence somewhat undermined the prominent position of these relatively coarse measures [2]–[4]. New data are emerging from studies that combine advanced phenotyping techniques allowing mean particle diameter measurement [5], [6], assessment of C and TG concentration down to particular lipoprotein subfractions [7]–[9] or comprehensive lipidomic approaches [10] together with genetic, epigenetic [11] as well as genome-wide analyses. Several recent studies identified genetic polymorphisms contributing to variation in lipoprotein diameters in distinct geoethnic groups [6], [12] or patterns of lipoprotein subfraction distribution [13]–[16]. As the TG and C content across the lipoprotein spectrum is substantially influenced by both genetic factors and environmental effects including diet [17]–[19] and medication [20]–[22], mammalian models have been successfully used for reduction of complexity in the detailed analyses of their genetic determinants [23], [24]. Already in 1996, an association study of the major lipoprotein subfractions was performed in 30 recombinant inbred HXB BXH strains derived from spontaneously hypertensive (SHR) and congenic Brown Norway (BN-*Lx*) rat strains [25]. The animals were fed a diet with 5% olive oil and 2% cholesterol for four weeks and the subsequent association analysis using 534 microsatellite markers identified loci on rat chromosomes 4, 19 and 20 linked to HDL2-C and phospholipids [25]. One of the strains from the original HXB/BXH set, the BXH2 strain, served as a progenitor of the PXO recombinant inbred strain panel together with chromosome 8 congenic strain SHR.*Lx* [26], [27]. Diets with high content of sucrose are well established to induce dyslipidemia (and insulin resistance) as recently reviewed by Gibson et al. [28]. We and others have established particular differences in the sensitivity to high-sucrose diet among the three strains contributing to genomic pool of both HXB/BXH and PXO recombinant inbred strains. While the spontaneously hypertensive rat SHR/OlaIpcv [29] and the polydactylous rat PD/Cub [30], [31] are quite sensitive both to hyperlipidemic and diabetogenic effects of HSD, the Brown Norway (BN) strain is less responsive in this respect [32], [33]. Furthermore, all the PXO strains carry the chromosome 8 region of PD/Cub origin that was shown to be responsive to HSD-induced dyslipidemia on both SHR and BN genomic backgrounds [33], [34]. In the current study, we performed genome-wide association of lipoprotein particle size, detailed distribution of C and TG into 20 distinct lipoprotein fractions and morphometric measures in the whole PXO set followed by *in silico* prioritization of candidate variants using dense map of over 20,000 single nucleotide polymorphisms and available whole genome sequences of SHR and BN rat strains.

## Materials and Methods

All experiments were performed in agreement with the Animal Protection Law of the Czech Republic (311/1997) which is in compliance with the European Community Council recommendations for the use of laboratory animals 86/609/ECC and were approved by the Ethical Committee of the First Faculty of Medicine of the Charles University.

### Rat strains

Adult (4-months old) male rats of 15 PXO strains (PXO1/Cub, Rat Genome Database (RGD) ID 2307138, n = 11; PXO2/Cub, RGD ID 2307356, n = 11; PXO3-1/Cub, RGD ID 2307355, n = 10; PXO3-2/Cub, RGD ID 2307116, n = 9; PXO4/Cub, RGD ID 2307135, n = 9; PXO5-1/Cub, RGD ID 2307128, n = 11; PXO5-2/Cub, RGD ID 2307117, n = 10; PXO6-1/Cub, RGD ID 2307137, n = 10; PXO6-2/Cub, RGD ID 2307125, n = 8; PXO6-3/Cub, RGD ID 2307130, n = 10; PXO7-1/Cub, RGD ID 2307119, n = 8; PXO8-1/Cub, RGD ID 2307122, n = 10; PXO8-2/Cub, RGD ID 2307131, n = 13; PXO9/Cub, RGD ID 2307118, n = 9; PXO10/Cub, RGD ID 2307132, n = 12)) as well as the progenitor strains SHR-*Lx* (RGD ID 61106, n = 13) and BXH2/Cub (BXH2 hereafter, RGD ID 2307121, n = 18) were utilized in the current study. All abovementioned strains were originally derived and kept since at the animal facility of Institute of Biology and Medical Genetics, Fist Faculty of Medicine, Charles University in Prague [27].

### Experimental protocol

Animals were held under temperature and humidity controlled conditions on 12 h/12 h light-dark cycle. At all times, the animals had free access to food and water. At the age of 4 months, male rats of 15 PXO strains (n = 8–13/strain) and the progenitor strains SHR-*Lx* (n = 13) and BXH2 (n = 18) were fed a high-sucrose diet (HSD, proteins (19.6 cal%), fat (10.4 cal%), carbohydrates (sucrose, 70 cal%) and balanced levels of micronutrients) for 1 week. Blood samples were drawn after overnight fasting from the tail vein. Using high performance liquid chromatography (HPLC), triglyceride (TG) and cholesterol (C) concentrations in 20 lipoprotein fractions and the size of major classes of lipoprotein particles were determined as described previously [9], [22]. Then the rats were sacrificed and the weights of heart, liver, kidneys, adrenals, epididymal and retroperitoneal fat pads were determined. The detailed results of all measurements are provided in **Tables S1–S6**.

### Genomic characterization

The rat genomic DNA was isolated from the tail incision samples using a modified phenol extraction method. Polymorphic microsatellite loci were amplified by PCR using conditions optimized for each marker. The PCR products were separated on polyacrylamide (7–10%) or agarose (2–4%) gels, stained by ethidium bromide and visualized using Syngene’s G-Box. We scanned over 1000 microsatellite markers for polymorphisms between the two progenitor strains. Subsequently, we selected markers polymorphic between the progenitor strains for genotyping all 15 PXO recombinant inbred strains. Total of 704 microsatellite markers were used for association analysis. Once the QTLs were identified, we determined *in silico* the extent of the haplotype blocks using the map of >20,000 single nucleotide polymorphisms, which has been recently made available by STAR consortium (http://www.snp-star.eu/) [35].

### Association and *in silico* analyses

The association analyses were performed using MapManagerQTX v0.30 [36]. The genome-wide significance was determined by permutation approach implemented in MapManagerQTX v0.30 (2000 permutations per trait). In order to identify the regions of the human genome syntenic to the identified QTLs, we have utilized the Virtual Comparative Map software tool (http://www.animalgenome.org/VCmap/). Then we have overlapped these regions with genomic positions of the significant loci reported in human genome-wide association studies (extracted from the Catalog of Published Genome-Wide Association Studies, available at: http://www.genome.gov/gwastudies/ [37]) for relevant phenotypes (in alphabetical order: Apolipoprotein Levels; Biochemical measures (*lipid-related*); Cardiovascular disease risk factors; Cholesterol; Cholesterol and Triglycerides; Cholesterol, total; HDL Cholesterol - Triglycerides (HDLC-TG); HDL cholesterol; Hypertriglyceridemia; LDL (oxidized); LDL cholesterol; Lipid metabolism phenotypes; Lipid traits; Metabolic syndrome (bivariate traits); Metabolic traits (*lipid-related*); Phospholipid levels (plasma); Quantitative traits (*lipid-related*); Sphingolipid levels; Triglycerides; Triglycerides-Blood Pressure (TG-BP).

### Statistical analysis

All statistical analyses were performed using STATISTICA 10 CZ. Unpaired Student’s t-test was used for comparison of metabolic and morphometric traits of the two progenitor strains. When comparing morphometric and biochemical variables in the whole PXO set, one-way ANOVA was used with STRAIN as major factor followed by post-hoc Tukey’s honest significance difference test for comparison of the specific pairs of variables. Null hypothesis was rejected whenever p<0.05.

## Results

### The morphometric profile of progenitors and the PXO set

**Figure 1:**
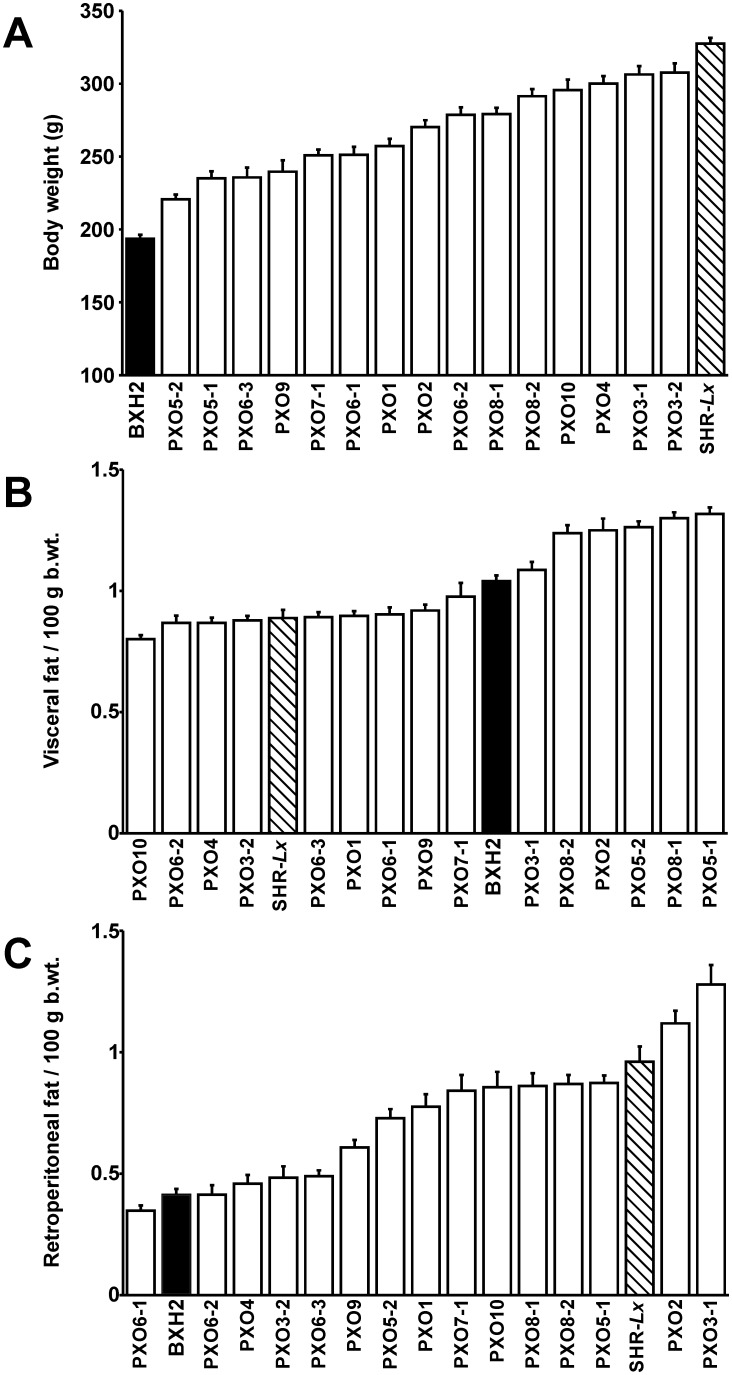
Strain distribution pattern for body weight (b.wt., panel A); visceral fat weight/100 g b.wt. (panel B) and retroperitoneal fat weight/100 g b.wt. (panel C), ordered by their means. Values are shown as means ± S.E.M. for adult male rats of progenitor strains BXH2 (n = 18, black bar), SHR-*Lx* (n = 13, striped bar) and all 15 PXO recombinant inbred strains (n = 8–13/strain, white bars).

The two progenitor strains showed significant differences in both morphometric and overall lipid profiles (**Table 1**). SHR-*Lx* rats were 69% heavier, displaying comparable amounts of visceral fat, however, more than twice as much of retroperitoneal fat per 100 g body weight compared to HXB2. Within the PXO recombinant inbred panel, body weight was distributed between the values of progenitors, while the fat deposit relative weights displayed a transgressive variation (**Figure 1**) as did other organ weights (**Table S1**).

**Table 1:** Phenotypic comparison of BXH2 and SHR-*Lx* progenitor strains.

Trait	BXH2 (n = 18)	SHR-*Lx* (n = 13)	p
Body weight, g	194±3	328±4	**<0.0001**
Liver wt., g/100 g b.wt.	2.52±0.02	3.06±0.03	**<0.0001**
Heart wt., g/100 g b.wt.	0.37±0.02	0.41±0.02	*0.42*
Kidney wt., g/100 g b.wt.	0.63±0.01	0.62±0.01	*0.39*
Adrenals wt., g/100 g b.wt.	0.017±0.001	0.010±0.0003	**<0.001**
Testes wt., g/100 g b.wt.	1.28±0.03	0.97±0.01	**0.030**
EFP wt., g/100 g b.wt.	1.04±0.02	0.89±0.03	*0.08*
RFP wt., g/100 g b.wt.	0.41±0.02	0.96±0.06	**<0.001**
Total cholesterol, mg/dl	48.7±1.6	53.7±1.2	**0.027**
Total triglyceride, mg/dl	30.1±1.7	33.0±4.0	*0.05*
Free glycerol, mg/dl	4.18±0.19	4.70±0.29	*0.09*
VLDL size, nm	42.6±0.6	40.5±0.1	**0.020**
LDL size, nm	21.8±0.1	20.7±0.1	**<0.001**
HDL size, nm	12.6±0.1	13.0±0.1	**<0.001**

Basic phenotypic profile of **BXH2** *vs.* **SHR-** ***Lx*** rats after one week of high-sucrose diet feeding with p-values (significant p values in bold, non-significant in italics) for unpaired Student’s t-test. Values are shown as mean ± S.E.M.; b.wt.…body weight; EFP…epididymal fat pad; RFP…retroperitoneal fat pad; VLDL - very low-density lipoprotein, LDL - low density lipoprotein, HDL - high-density lipoprotein.

### The lipid profile of progenitors and the PXO set

**Figure 2:**
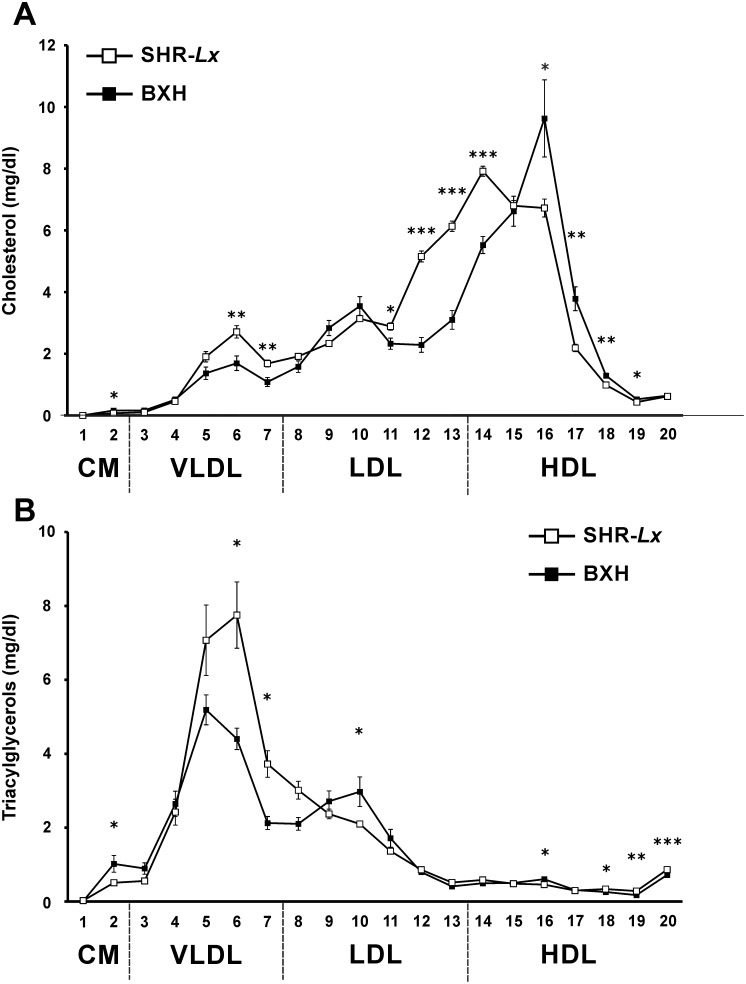
Fasting cholesterol (A) and triacylglycerol (B) content in 20 lipoprotein subfractions in SHR-*Lx* (squares) vs. BXH2 (diamonds) adult male rats after one week of high-sucrose diet. Within the graph, the significance levels of strain comparison by post-hoc Tukey’s HSD test are shown as follows: *…p<0.05; **…p<0.01; ***…p<0.001. The allocation of individual lipoprotein subfractions to major lipoprotein classes is shown in order of particle’s decreasing size from left to right. CM - chylomicron, VLDL - very low-density lipoprotein, LDL - low density lipoprotein, HDL - high-density lipoprotein.

**Figure 3:**
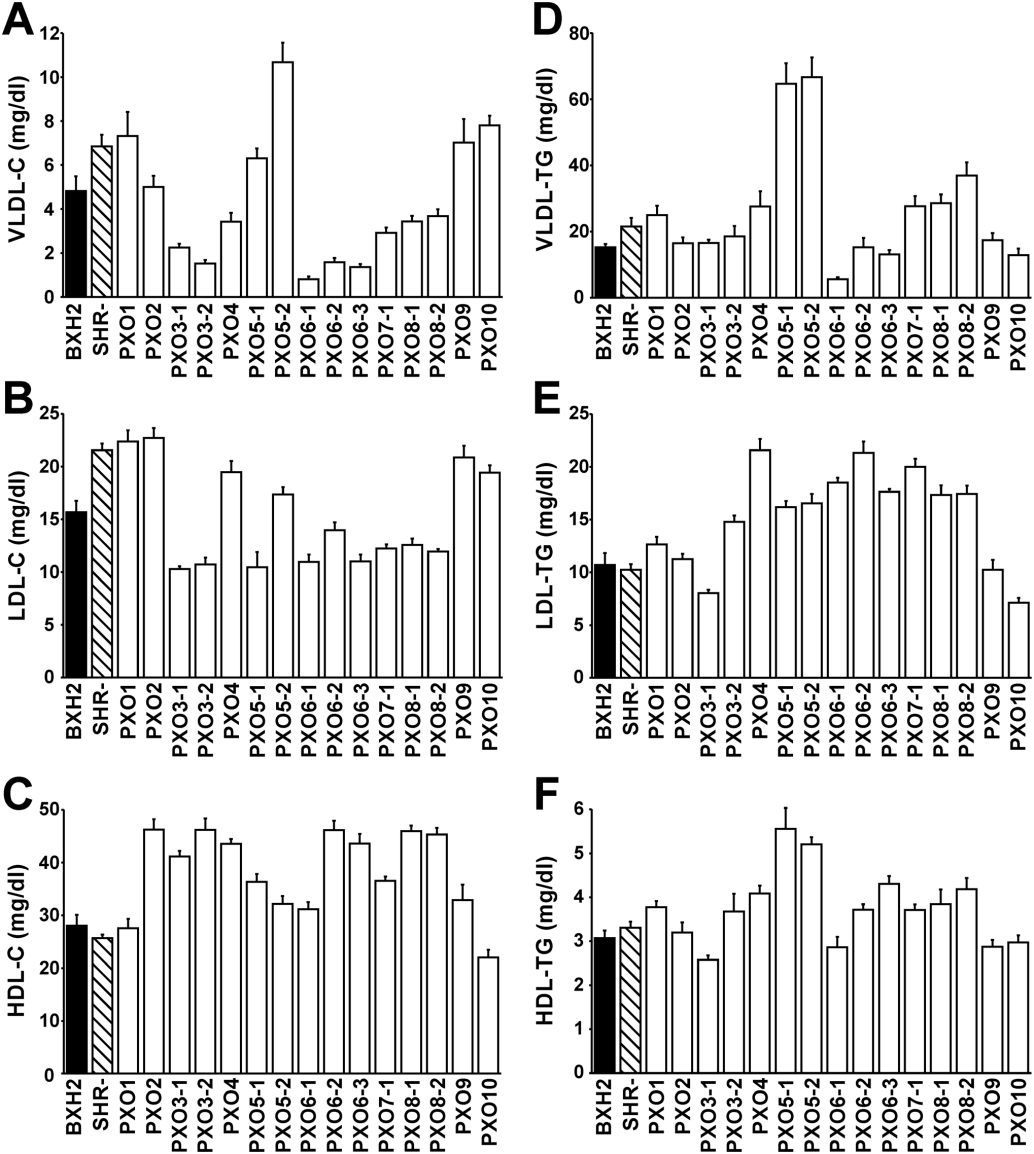
Strain distribution pattern for fasting concentrations of cholesterol (C) and triglyceride (TG) in very low-density lipoprotein (VLDL, panels A, D); low-density lipoprotein (LDL, panels B, E); high-density lipoprotein cholesterol (HDL, panels C, F). Values are shown as means ± S.E.M. for adult male rats of BXH2 (n = 18, black bar), SHR-*Lx* (n = 13, striped bar) and all 15 PXO recombinant inbred strains (n = 8–13/strain, white bars).

Concentration of total C was slightly higher in SHR-*Lx* compared to BXH2, while total TG did not differ between the progenitors (**Table 1**). However, the detailed assessment of the C and TG distribution into 20 lipoprotein fractions revealed a rather unfavorable profile in SHR-*Lx* rats (**Tables S5** and **S6**). Compared to BXH2, SHR-*Lx* had increased levels of TG and C in VLDL fraction and most significant cholesterol enrichment in the very small LDL particles (**Figure 2**). Except for very large and very small HDL, the HDL-C content was decreased in SHR-*Lx*. When assessing the lipid profile of the whole PXO panel, we observed a substantial gradient in C and TG concentrations within the major lipoprotein classes (**Figure 3**, **Tables S2 and S3**), particularly in VLDL (13-fold and 12-fold gradient across the PXO strains for C and TG, respectively). Combined with limited intra-strain variance of these measures this suggested actual segregation of alleles affecting the distribution of TG and C, making the studied traits amenable to genetic dissection in the PXO panel.

### The genome-wide association

We scanned over 1000 microsatellite markers for polymorphisms between SHR-*Lx* and BXH2 and subsequently we genotyped 704 polymorphic markers in all PXO recombinant inbred strains, evenly covering autosomes and X-chromosome (except for chromosomes 2 and 13 that are of SHR origin in both parental strains). Blocks of adjacent markers with identical segregation patterns were collapsed into the final strain distribution pattern (SDP). The complete SDP was used for association analysis of morphometric and lipid-related traits. Using interval mapping, we have identified nine loci showing suggestive or significant association to 13 studied traits (3 morphometric and 10 lipid-related loci, summarized in **Table 2**). Except for association signals for adiposity index and LDL-TG on chromosomes 3 and 12, PXO strains carrying the SHR-*Lx* (i.e. SHR) allele displayed significantly higher values of the linked traits, e.g. LDL-C (21.2±0.4 vs. 12.5±0.4 mg/dl in PXO strains with SHR allele vs. BXH2 (BN) allele in the *D3Rat50*-*D3Got19* block (**Table 2**).

**Table 2:** Suggestive and significant associations in the PXO recombinant inbred set.

Trait	Chr.	Flanking markers	LOD	%	SNP focusing(kbp)	genes	SHR/SHR	BN/BN	unit
LDL-C	3	D3Rat50-D3Got19	**6.3**	56%	16,845–20,626*	21	21.2±0.4	12.5±0.4	mg/dl
f12-C (v. small LDL)	3	D3Rat50-D3Got19	**4.7**	59%	16,845–20,626*	21	3.9±0.1	2.1±0.1	mg/dl
f8-C (large LDL)	3	D3Got175-D3Rat49	**5.8**	39%	20,496–22,532	1	2.2±0.1	0.9±0.1	mg/dl
f9-C (med. LDL)	3	D3Got175-D3Rat49	*4.4*	41%	20,496–22,532	1	3.2±0.1	1.5±0.1	mg/dl
f12-C (v. small LDL)	3	D3Got175-D3Rat49	**4.7**	67%	20,496–22,532	1	4.1±0.1	2.1±0.1	mg/dl
f8-C (large LDL)	3	D3Rat100-D3Rat66	**5.8**	19%	36,500–42,539	22	2.2±0.1	0.9±0.1	mg/dl
f8-TG (large LDL)	3	D3Rat100-D3Rat66	*3.4*	5%	36,500–42,539	22	2.5±0.2	3.6±0.2	mg/dl
f9-C (med. LDL)	3	D3Rat100-D3Rat66	*4.4*	12%	36,500–42,539	22	3.2±0.1	1.5±0.1	mg/dl
VLDL-C	4	D4Rat156-D4Mit2	*3.6*	60%	48,953–50,153	5	7.8±0.4	2.8±0.2	mg/dl
f5-C (large VLDL)	4	D4Rat156-D4Mit2	*4.1*	61%	48,953–50,153	5	2.3±0.1	0.8±0.1	mg/dl
f6-C (med. VLDL)	4	D4Rat156-D4Mit2	*3.7*	64%	48,953–50,153	5	2.8±0.1	1.1±0.1	mg/dl
Liver wt	7	D7Rat206-D7Got52	*3.8*	28%	74,492–76,763	9	8.53±0.16	6.22±0.07	g
Kidney wt	7	D7Rat206–D7Got52	**4.9**	36%	74,492–76,763	9	2.00±0.03	1.44±0.01	g
HDL-C size	7	D7Rat20-D7Rat15	*3.5*	51%	100,067–107,086*	20	12.76±0.02	12.43±0.03	nm
Adiposity index	8	D8Rat26-D8Arb13	*4.1*	11%	79,537–87,548*	39	1.01±0.02	1.11±0.05.	g/100 g b.wt
f12-C (v. small LDL)	11	Sst-D11Rat43	**4.7**	63%	79,418–84,879*	65	3.8±0.1	2.1±0.1	mg/dl
LDL-TG	12	D12Wox9-D12Got42	*3.5*	53%	17,168–20,718*	42	10.8±0.5	17.7±0.5	mg/dl

Summary of quantitative trait loci identified in the PXO recombinant inbred rat panel. The letter “f” followed by a number (e.g. f5) corresponds to a particular fraction of lipoproteins as depicted in **Figure 1**. SNP (single nucleotide polymorphism) focusing column denotes the **position of the QTL on the chromosome (**Chr.) according to the Rat Genome V3.4 Assembly. % - percentage of trait variance explained by QTL, C – cholesterol, TG – triacylglycerols; LOD – likelihood of odds score (**bold** for genome-wide significance, *italics* for suggestive association); VLDL - very low-density lipoprotein; LDL - low density lipoprotein; HDL - high-density lipoprotein; wt – weight; adiposity index – calculated as epididymal (visceral) fat weight per 100 g of body weight; *…region contains known CNV distinct between SHR and BN-*Lx* [39]. The total number of genes within the QTL region is shown. Mean values ± SEM are shown for PXO strains carrying the SHR-Lx (SHR/SHR) allele vs. those carrying the BXH2/Cub allele (BN/BN) for the given trait and locus.

### *In silico* analyses

In order to narrow down the genomic extent of the identified QTLs, we have retrieved SNP genotypes of progenitors and PXO strains for the identified associated regions using the SNPlotyper tool (http://snplotyper.mcw.edu/, **Table 2**) [35]. In some cases, we were able to significantly reduce the extent of the particular haplotype block (e.g. for VLDL-C QTL on chromosome 4 from 8.7 Mb to just 1.1 Mb containing only 4 genes, i.e. by 86%), in other cases the information provided by microsatellites was almost identical to that by SNPs (e.g. the LDL-C QTL on chromosome 3 was shortened by 350 kb, i.e. by 8%). In the next step, we utilized the Variant Visualizer resource provided by Rat Genome Database (http://rgd.mcw.edu/rgdweb/front/select.html) to identify highly conserved variants between SHR and BN genome sequences [38] among the genes captured within the narrowed QTLs as potential candidates for further targeted studies. These genes represent primary candidates for the identified QTLs. At the same time, we have searched for the presence of copy number variants distinct in SHR an BN genomes within the QTLs [39].

The strongest association signal was detected for cholesterol content in total LDL and very small LDL particles on rat chromosome 3. Among 21 protein-coding genes in the associated segment, seven showed non-synonymous variants between the SHR and BN alleles: ring finger and CCCH-type domains 2 (*Rc3h2*), G protein-coupled receptor 21 (*Gpr21*), RAB GTPase activating protein 1 (*Rabgap1*, there was another variant within 5′-untranslated region of this gene), crumbs homolog 2– Drosophila (*Crb2*), similar to cis-Golgi matrix protein GM130 (LOC690458), Golgin subfamily A member 1 (*Golga1*) and 40 s ribosomal protein S17-like (LOC100362666). Four intronic variations were found within spermatid perinuclear RNA binding protein (*Strbp*).

A distinct locus on chromosome 3 was linked to cholesterol concentration in large, medium and very small LDL particles. The whole genomic block spanning about 2 Mbp overlaps with a single gene, low density lipoprotein receptor-related protein 1B (*Lrp1b*). While no mutation putatively changing an amino-acid was found when comparing the sequences of the two progenitors, there were >20 intronic differences spread across the associated region as well as ten and five distinct variants within 5′- and 3′- untranslated regions (UTR) of this gene, respectively. Five genes within the third locus on chromosome 3 linked to cholesterol and triacylglycerol concentrations in large LDL particles and cholesterol in medium LDL have distinct non-synonymous variants in SHR: hypothetical protein LOC680314, tetratricopeptid repeat, ankyrin repeat and coiled-coil containing 1 (*Tanc1*, additional 3 differences were found in 5′-UTR), bromodomain adjacent to zinc finger domain, 2B (*Baz2b,* within 5′-UTR of this gene there were 9 other polymorphisms), lymphocyte antigen 75 (*Ly75*) and phospholipase A2 receptor 1 (*Pla2r1*).

The quantitative trait locus identified on chromosome 4 shows association to overall concentration of cholesterol in VLDL as well as in medium and large VLDL particles. Spanning just over 1 Mbp, it contains two genes with predicted aminoacid changes in SHR, the protein tyrosine phosphatase, receptor-type, Z polypeptide 1 (*Ptprz1*) and aminoadipate – semialdehyde synthase (*Aass*). More than 10 intronic variants were found in the Ca++-dependent secretion activator 2 (*Cadps2*) gene.

Chromosome 7 was a site of two association hits; the one associated with morphometric traits for liver and kidney weight encompasses 5 genes with differences between SHR and BN strains [frizzled family receptor 6 (*Fzd6*, 1 synonymous and 2 nonsynonymous variants in SHR), dendrocyte expressed seven transmembrane protein (*Dcstamp*, 1 nonsynonymous variant and 4 SNPs in introns), regulating synaptic membrane exocytosis 2 (*Rims2,* 9 SNPs in introns), non-synonymous variants were found also in dihydropyrimidinase (*Dpys*) and zinc finger protein, FOG family member 2 (*Zfpm2*)]. This region actually overlaps with previously identified salt-loaded kidney mass in Dahl salt-sensitive rats [40]. The genes containing non-synonymous variants distinguishing the SHR and BN alleles within the second locus on chromosome 7 linked to size of HDL cholesterol particles were transmembrane protein 71 (*Tmem71*), thymoglobulin (*Tg*) and WNT1 inducible signaling pathway protein 1 (*Wisp1*).

The only significant association for adiposity index is located on chromosome 8. Although the related block of SDP spans about 8 Mbp, there are only 10 genes with *in silico* evidence of non-synonymous variants in SHR: myosin VC (*Myo5c*), family with sequence similarity 83, member B (*Fam83b*), tubulointerstitial nephritis antigen (*Tinag*), kelch-like family member 31 (*Klhl31*), intestinal cell (MAK-K) kinase (*Ick*), collagen type 12, alpha 1 (*Col12a1*), gamma subunit-like (*Sec61*, LOC100363239), filamin A interacting protein 1 (*Filip1*), SUMO1/sentrin specific peptidase 6 (*Senp6*) and interphotoreceptor matrix proteoglycan 1 (*Impg1*). Exceptionally in the context of the current study, it is the BN allele that drives increased adiposity. We have previously identified similar effect of this BN locus in a PD/Cub x BN intercross [30], mesenteric fat amount QTL was found in The Otsuka Long–Evans Tokushima Fatty (OLETF) rat [41].

The significant QTL for cholesterol concentration in very small LDL particles on chromosome 11 includes eight genes (out of 65 present in the associated region) showing genetic variation between SHR and BN: diacylglycerol kinase gamma (*Dgkg*), enoyl-Coa, hydratase/3-hydroxyacyl Coa dehydrogenase (*Ehhadh*), ephrin type-B receptor 3 (*Ephb3*), alpha-1,3-mannosyltransferase (*Alg3*), ATP-binding cassette, subfamily C (CFTR/MRP), member 5 (*Abcc5*), kelch-like family member 6 (*Klhl6*), Ig lambda chain V-VI region SUT-like (LOC680311) and septin 5 (*Sept5*).

The sole association of overall triacylglycerol concentration in LDL maps to chromosome 12 with five genes showing sequence variants between SHR and BN leading to aminoacid change: sprouty homolog 3, Drosophila (*Spry3*), ribosomal protein L31-like 4 (*Rpl31l4*, with one additional intronic SNP), similar to family with sequence similarity 55, member C (RGD1564217), vomeronasal 2 receptor 63 (*Vom2r63*) and methylphosphate capping enzyme (*Mepce*). Furthermore, distinct SHR alleles were identified for zinc finger with KRAB and SCAN domains 1 (*Zkscan1*, 2 SNPs in 5′-UTR, 2 SNPs in introns), ArfGAP with FG repeats 2 (*Agfg2*, 4 SNPs in 5′-UTR), stromal antigen 3 (*Stag3*, 2 intronic SNPs) and cytochrome P450, family 3, subfamily a, polypeptide 9 (*Cyp3a9*, 1 SNP in intron). This region has not been yet linked to triacylglycerol levels in the rat studies, but several cholesterol QTLs map to this region [42], [43].

## Discussion

This study represents one of the first scans of genomic determinants of detailed distribution of cholesterol and triacylglycerols into lipoprotein fractions in the rat. The PXO recombinant inbred set represents a genetically fixed system with segregating alleles of SHR, i.e. hypertensive model of metabolic syndrome; limited portion of chromosome 8 of polydactylous rat PD/Cub, a model of metabolic syndrome without hypertension [44] and normotensive and normolipidemic Brown Norway (BN/Cub) strain [27]. The studies of PXO and the original HXB BXH recombinant inbred strain panels have repeatedly provided crucial insights into the genomic architecture of human complex cardiometabolic traits [26], [45], [46] and has served as a platform for the “proof-of-concept” type of studies, e.g. for genetical genomics [47].

Majority of significant associations in our study were found for lipid concentrations in subclasses of LDL lipoproteins, three of them mapped to distinct regions of rat chromosome 3. So far, two rat QTLs for total cholesterol levels were reported in the overlapping regions, one in a metabolic syndrome model – the Wistar Ottawa Karlsburg W (WOKW) rat [48] [49] and the second in an intercross of Dahl S x R rats transgenic for human cholesteryl ester transfer protein (*CETP*) [50]. A single gene, orphan G protein coupled receptor *Gpr21* stands out as possible candidate for the most significant observed association of cholesterol in total and very small LDL, although no direct connection to lipid metabolism is currently known. The *Gpr21* gene is widely expressed [51] and has been shown to possess critical function in coordinating macrophage proinflammatory activity in the context of obesity-induced insulin resistance [52]. *Gpr21*-knockout mice were shown to have improved insulin sensitivity and lower triacylglycerol content in liver when fed high-fat diet [52].

Altogether, we have identified three significant associations for cholesterol concentration in very small LDL particles. It has been repeatedly shown that small, dense LDL are clearly connected with metabolic syndrome, type 2 diabetes and fatty liver [53] [54] While the unfavorable lipidemic profile is greatly influenced by environment, particularly lifestyle, possible identification of sensitizing genetic variants may eventually allow early identification of individuals at enhanced risk of coronary artery disease and related conditions. We have also compared the complete set of genes present in the regions with identified association to any of the followed traits with the repository of results coming from human genome-wide association studies (http://www.genome.gov/gwastudies/) [37]. We did not identify any directly corresponding associations (see Methods) between human GWAS and the putative variants in SHR genome. This is not surprising given the rather subtle nature in risk increase conferred by most SNPs identified in this type of human studies. On the other hand, variants in several genes including *LRP1B* and *DGKG*, associated with cholesterol concentrations in very small LDL particles in our study, were associated with body mass index and weight human studies [55], [56]. LRP1B belongs to the LDL receptor family and its mutations are often found in human cancers [57]. Furthermore, polymorphisms in the *LRP1B* gene were found to be associated with insulin resistance [58] and preeclampsia [59] in human studies. Also, *Lrp1b* was identified as significant member of genome-wide signatures of abdominal fatness in chicken [60]. The importance of identification of very narrow QTL encompassing only part of single gene (*Lrp1b*) is enhanced by *in silico* data suggesting presence of five non-synonymous variants distinct in SHR vs. BN within the QTL. Since the expression of *LRP1B* was found to be relatively low compared to the homologous *LRP1* (and due to its localization mainly in brain, skeletal muscle and thyroid gland) it was suggested that LRP1b does not play a major role in the clearing of circulating lipoproteins from plasma, on the other hand, its capability of binding apoE-containing ligands has been demonstrated [61]. Detailed study of this large gene is needed to validate causality of the ascertained variants in SHR.

The third region on rat chromosome 3 identified in the current study affecting cholesterol and triacylglycerol concentrations in LDL harbors three genes with non-synonymous mutations in SHR with indirect supportive evidence from other studies. The variants in *BAZB2* gene have been shown as prime candidates for sudden cardiac death in GWAS meta-analysis performed in individuals with European ancestry [62] while *LY75* was identified as a prime expression SNP for type 2 diabetes [63] and significant changes in *LY75* expression due to aberrant methylation was found in human dilated cardiomyopathy [64]. The *Pla2r1* functions as a receptor of the enzyme secretory phospholipase A2, which in turn catalyzes the hydrolysis of phospholipids. The *PLA2R1* gene has been proposed as a candidate for coronary artery disease in subjects with normal levels of total cholesterol [65] and very recently the *Pla2r*-deficient mice were shown to be susceptible to cardiac rupture after myocardial infarction [66].

While two regions associated in our study to levels of cholesterol in VLDL and very small LDL (chromosomes 4 and 11, respectively) were not yet linked to cholesterol in other rat studies, there are several reports showing their association to triacylglycerol concentrations in WOKW [67], SHR [68] [69] and Dahl salt-sensitive rat models [40]. One of the limitations of the current study is the incomplete coverage of all autosomes, as chromosomes 2 and 13 are of SHR origin in both parental strains. Therefore, possibly existing polymorphisms with influence on lipid traits residing at the two chromosomes could not be identified by our association analysis. To that end, we have previously derived dedicated chromosome 2 congenic strain and reported the effect of SHR alleles on TG and C distribution into lipoprotein fractions [70]. Another limitation is represented by the fact that all measurements were performed under high-sucrose diet-feeding and therefore the identified associations may be related only to this particular nutritional condition (i.e. the “nutrigenetic” layer of the dynamic genetic architecture [30] of lipid profile). In summary, we have combined detailed phenotyping of a unique rat strain panel together with classical and *in silico* approaches to identify novel candidates for genetic determinants of cholesterol and triacylglycerol distribution into lipoprotein subfractions. The process of identification of candidate genes and positional cloning of causal variants is substantially streamlined thanks to the availability of comprehensive genotype data and full genomic sequences of the spontaneously hypertensive and Brown Norway rat strains.

## Supporting Information

Table S1 **Morphometric profile of the PXO recombinant inbred strain panel and its progenitor strains, BXH2/Cub and SHR-** ***Lx*** **.**
(PDF)

Table S2 **Cholesterol concentration in major lipoprotein fractions and free glycerol in the PXO recombinant inbred strain panel and its progenitor strains, BXH2/Cub and SHR-** ***Lx*** **.**
(PDF)

Table S3 **Triacylglycerol concentration in major lipoprotein fractions in the PXO recombinant inbred strain panel and its progenitor strains, BXH2/Cub and SHR-** ***Lx*** **.**
(PDF)

Table S4 **Profile of sizes of major lipoprotein particles in the PXO recombinant inbred strain panel and its progenitor strains, BXH2/Cub and SHR-** ***Lx*** **.**
(PDF)

Table S5 **Cholesterol concentration in individual lipoprotein fractions l in the PXO recombinant inbred strain panel and its progenitor strains, BXH2/Cub and SHR-** ***Lx*** **.**
(PDF)

Table S6 **Triacylglycerol concentration in individual lipoprotein fractions l in the PXO recombinant inbred strain panel and its progenitor strains, BXH2/Cub and SHR-** ***Lx*** **.**
(PDF)
