# Supplementary material for: Genomic Determinants of Triglyceride and Cholesterol Distribution into Lipoprotein Fractions in the Rat
Source: PLoS One. 2014 Oct 8;9(10):e109983. doi: 10.1371/journal.pone.0109983 (PMC4190321; doi:10.1371/journal.pone.0109983)
Supplement: Table S1 — Morphometric profile of the PXO recombinant inbred strain panel and its progenitor strains, BXH2/Cub and SHR- Lx . (PDF) [file pone.0109983.s001.pdf]

	Body weight (g)			Heart weight (g/100g b.wt.)			Liver weight (g/100g b.wt.)			Kidney weight (g/100g b.wt.).			Adrenals weight (g/100g b.wt.)			Testes weight (g/100g b.wt.)			Epididymal fat pad weight (g/100g b.wt.)			Retroperitoneal fat pad weight (g/100g b.wt.)	
**STRAIN**	**mean**	**SEM**	**STRAIN**	**mean**	**SEM**	**STRAIN**	**mean**	**SEM**	**STRAIN**	**mean**	**SEM**	**STRAIN**	**mean**	**SEM**	**STRAIN**	**mean**	**SEM**	**STRAIN**	**mean**	**SEM**	**STRAIN**	**mean**	**SEM**
**BXH2**	193.7	*2.8*	**PXO3-2**	0.31	*0.01*	**PXO6-2**	2.37	*0.03*	**PXO8-2**	0.55	*0.01*	**SHR-Lx**	0.010	*0.0003*	**PXO2**	0.77	*0.05*	**PXO10**	0.80	*0.02*	**PXO6-1**	0.35	*0.02*
**PXO5-2**	220.6	*3.3*	**PXO6-3**	0.32	*0.00*	**PXO6-1**	2.38	*0.03*	**PXO8-1**	0.56	*0.004*	**PXO2**	0.012	*0.0013*	**PXO10**	0.91	*0.08*	**PXO6-2**	0.87	*0.03*	**BXH2**	0.41	*0.03*
**PXO5-1**	235.1	*4.8*	**PXO7-1**	0.33	*0.01*	**PXO2**	2.48	*0.03*	**PXO6-2**	0.56	*0.01*	**PXO8-2**	0.012	*0.0004*	**SHR-Lx**	0.97	*0.01*	**PXO4**	0.87	*0.02*	**PXO6-2**	0.41	*0.04*
**PXO6-3**	235.6	*6.8*	**PXO10**	0.34	*0.01*	**PXO9**	2.49	*0.05*	**PXO2**	0.57	*0.01*	**PXO9**	0.013	*0.0010*	**PXO3-2**	0.99	*0.02*	**PXO3-2**	0.88	*0.02*	**PXO4**	0.46	*0.03*
**PXO9**	245.0	*6.2*	**PXO1**	0.35	*0.02*	**PXO8-1**	2.49	*0.03*	**PXO5-1**	0.58	*0.01*	**PXO4**	0.013	*0.0008*	**PXO6-2**	0.99	*0.02*	**SHR-Lx**	0.89	*0.03*	**PXO3-2**	0.48	*0.04*
**PXO7-1**	250.9	*3.8*	**PXO6-2**	0.37	*0.02*	**BXH2**	2.52	*0.02*	**PXO7-1**	0.58	*0.01*	**PXO3-1**	0.014	*0.0005*	**PXO4**	1.01	*0.01*	**PXO6-3**	0.89	*0.02*	**PXO6-3**	0.49	*0.02*
**PXO6-1**	251.2	*5.5*	**PXO5-1**	0.37	*0.01*	**PXO8-2**	2.53	*0.02*	**PXO6-3**	0.59	*0.01*	**PXO5-1**	0.014	*0.0005*	**PXO3-1**	1.01	*0.04*	**PXO1**	0.90	*0.02*	**PXO9**	0.61	*0.03*
**PXO1**	257.2	*5.2*	**PXO4**	0.37	*0.01*	**PXO5-1**	2.55	*0.02*	**PXO1**	0.59	*0.01*	**PXO1**	0.014	*0.0008*	**PXO8-1**	1.06	*0.01*	**PXO6-1**	0.90	*0.03*	**PXO5-2**	0.73	*0.04*
**PXO2**	270.2	*4.7*	**PXO3-1**	0.37	*0.01*	**PXO4**	2.60	*0.03*	**PXO9**	0.60	*0.01*	**PXO7-1**	0.015	*0.0005*	**PXO8-2**	1.08	*0.02*	**PXO9**	0.92	*0.02*	**PXO1**	0.78	*0.05*
**PXO6-2**	278.6	*5.2*	**SHR-Lx**	0.37	*0.01*	**PXO6-3**	2.61	*0.04*	**PXO6-1**	0.60	*0.01*	**PXO8-1**	0.015	*0.0008*	**PXO6-3**	1.08	*0.02*	**PXO7-1**	0.98	*0.06*	**PXO7-1**	0.84	*0.06*
**PXO8-1**	279.1	*4.2*	**PXO8-2**	0.37	*0.01*	**PXO3-2**	2.62	*0.04*	**PXO3-2**	0.60	*0.01*	**PXO6-3**	0.015	*0.0011*	**PXO9**	1.11	*0.02*	**BXH2**	1.04	*0.02*	**PXO10**	0.86	*0.06*
**PXO8-2**	291.4	*4.8*	**PXO5-2**	0.38	*0.02*	**PXO1**	2.62	*0.02*	**PXO5-2**	0.60	*0.01*	**PXO10**	0.016	*0.0005*	**PXO1**	1.12	*0.02*	**PXO3-1**	1.09	*0.03*	**PXO8-1**	0.86	*0.04*
**PXO10**	295.6	*7.2*	**PXO2**	0.39	*0.01*	**PXO5-2**	2.65	*0.02*	**SHR-Lx**	0.62	*0.01*	**PXO3-2**	0.017	*0.0003*	**PXO6-1**	1.14	*0.04*	**PXO8-2**	1.24	*0.03*	**PXO8-2**	0.87	*0.03*
**PXO4**	300.1	*5.1*	**PXO9**	0.40	*0.01*	**PXO10**	2.72	*0.03*	**BXH2**	0.63	*0.01*	**BXH2**	0.017	*0.0008*	**PXO5-1**	1.15	*0.02*	**PXO2**	1.25	*0.05*	**PXO5-1**	0.87	*0.03*
**PXO3-1**	306.3	*5.1*	**PXO6-1**	0.41	*0.02*	**PXO7-1**	2.73	*0.03*	**PXO4**	0.63	*0.01*	**PXO6-2**	0.017	*0.0007*	**PXO5-2**	1.24	*0.01*	**PXO5-2**	1.26	*0.02*	**SHR-Lx**	0.96	*0.06*
**PXO3-2**	307.6	*5.8*	**BXH2**	0.42	*0.02*	**PXO3-1**	2.75	*0.03*	**PXO3-1**	0.63	*0.01*	**PXO5-2**	0.019	*0.0004*	**PXO7-1**	1.25	*0.01*	**PXO8-1**	1.30	*0.02*	**PXO2**	1.12	*0.05*
**SHR-*Lx***	327.8	*4.1*	**PXO8-1**	0.42	*0.03*	**SHR-Lx**	3.06	*0.02*	**PXO10**	0.73	*0.01*	**PXO6-1**	0.020	*0.0006*	**BXH2**	1.30	*0.03*	**PXO5-1**	1.32	*0.03*	**PXO3-1**	1.28	*0.07*

Supplementary Table S1. **Morphometric profile of the PXO recombinant inbred strain panel and its progenitor strains, BXH2/Cub and SHR-Lx. b.wt. = body weight.**
