# Supplementary material for: Genomic Determinants of Triglyceride and Cholesterol Distribution into Lipoprotein Fractions in the Rat
Source: PLoS One. 2014 Oct 8;9(10):e109983. doi: 10.1371/journal.pone.0109983 (PMC4190321; doi:10.1371/journal.pone.0109983)
Supplement: Table S2 — Cholesterol concentration in major lipoprotein fractions and free glycerol in the PXO recombinant inbred strain panel and its progenitor strains, BXH2/Cub and SHR- Lx . (PDF) [file pone.0109983.s002.pdf]

	Total cholesterol [mg/dL]			CM cholesterol [mg/dL]			VLDL cholesterol [mg/dL]			LDL cholesterol [mg/dL]			HDL cholesterol [mg/dL]			Free glycerol [mg/dL]	
**STRAIN**	**mean**	**SEM**	**STRAIN**	**mean**	**SEM**	**STRAIN**	**mean**	**SEM**	**STRAIN**	**mean**	**SEM**	**STRAIN**	**mean**	**SEM**	**STRAIN**	**mean**	**SEM**
**PXO6-1**	42.9	*1.7*	**PXO6-1**	0.01	*0.003*	**PXO6-1**	0.81	*0.12*	**PXO3-1**	10.28	*0.28*	**PXO10**	22.00	*1.47*	**PXO6-1**	3.34	*0.15*
**BXH2**	48.7	*1.6*	**PXO6-3**	0.02	*0.003*	**PXO6-3**	1.36	*0.14*	**PXO5-1**	10.45	*1.44*	**SHR-Lx**	25.67	*0.67*	**PXO3-1**	3.37	*0.21*
**PXO10**	49.4	*2.0*	**PXO6-2**	0.02	*0.01*	**PXO3-2**	1.52	*0.16*	**PXO3-2**	10.72	*0.65*	**PXO1**	27.54	*1.80*	**PXO3-2**	3.40	*0.15*
**PXO7-1**	51.7	*1.0*	**PXO3-2**	0.02	*0.003*	**PXO6-2**	1.58	*0.20*	**PXO6-1**	10.95	*0.71*	**BXH2**	28.01	*2.08*	**PXO1**	3.73	*0.22*
**PXO5-1**	53.3	*2.4*	**PXO7-1**	0.02	*0.01*	**PXO3-1**	2.26	*0.17*	**PXO6-3**	11.02	*0.62*	**PXO6-1**	31.15	*1.38*	**PXO4**	3.84	*0.19*
**PXO3-1**	53.7	*1.3*	**PXO3-1**	0.03	*0.003*	**PXO7-1**	2.91	*0.25*	**PXO8-2**	11.95	*0.24*	**PXO5-2**	32.16	*1.52*	**PXO2**	4.03	*0.19*
**SHR-Lx**	54.2	*1.3*	**PXO4**	0.03	*0.01*	**PXO4**	3.43	*0.40*	**PXO7-1**	12.24	*0.39*	**PXO9**	32.90	*2.96*	**PXO7-1**	4.08	*0.11*
**PXO6-3**	56.0	*1.6*	**PXO8-2**	0.05	*0.01*	**PXO8-1**	3.44	*0.25*	**PXO8-1**	12.56	*0.61*	**PXO5-1**	36.37	*1.47*	**PXO6-2**	4.12	*0.21*
**PXO1**	57.4	*1.4*	**PXO8-1**	0.06	*0.01*	**PXO8-2**	3.68	*0.31*	**PXO6-2**	13.96	*0.76*	**PXO7-1**	36.57	*0.82*	**BXH2**	4.18	*0.19*
**PXO3-2**	58.5	*2.7*	**SHR-Lx**	0.07	*0.01*	**BXH2**	4.82	*0.67*	**BXH2**	15.68	*1.08*	**PXO3-1**	41.19	*1.06*	**PXO8-2**	4.34	*0.28*
**PXO5-2**	60.6	*1.4*	**PXO9**	0.08	*0.02*	**PXO2**	5.00	*0.51*	**PXO5-2**	17.35	*0.72*	**PXO4**	43.61	*0.92*	**PXO9**	4.35	*0.19*
**PXO9**	60.9	*2.0*	**PXO10**	0.12	*0.02*	**PXO5-1**	6.31	*0.45*	**PXO10**	19.44	*0.68*	**PXO6-3**	43.66	*1.74*	**PXO8-1**	4.47	*0.20*
**PXO8-2**	61.0	*1.3*	**PXO1**	0.13	*0.02*	**SHR-Lx**	6.85	*0.53*	**PXO4**	19.47	*1.06*	**PXO8-2**	45.31	*1.29*	**PXO6-3**	4.65	*0.19*
**PXO6-2**	61.7	*2.6*	**PXO5-1**	0.16	*0.04*	**PXO9**	7.02	*1.08*	**PXO9**	20.88	*1.08*	**PXO8-1**	45.97	*1.02*	**SHR-Lx**	4.91	*0.23*
**PXO8-1**	62.0	*1.3*	**BXH2**	0.16	*0.04*	**PXO1**	7.32	*1.11*	**SHR-Lx**	21.56	*0.61*	**PXO6-2**	46.13	*1.76*	**PXO10**	5.10	*0.13*
**PXO4**	66.5	*1.3*	**PXO2**	0.17	*0.03*	**PXO10**	7.80	*0.44*	**PXO1**	22.37	*1.06*	**PXO3-2**	46.20	*2.14*	**PXO5-1**	5.45	*0.39*
**PXO2**	74.1	*1.8*	**PXO5-2**	0.42	*0.05*	**PXO5-2**	10.68	*0.89*	**PXO2**	22.72	*0.93*	**PXO2**	46.23	*1.97*	**PXO5-2**	5.84	*0.28*

Supplementary Table S2. Cholesterol concentration in major lipoprotein fractions and free glycerol in the PXO recombinant inbred strain panel and its progenitor strains, BXH2/Cub and SHR-Lx. CM - chylomicron, VLDL - very low-density lipoprotein, LDL - low density lipoprotein, HDL - high-density lipoprotein.
