# Supplementary material for: Genomic Determinants of Triglyceride and Cholesterol Distribution into Lipoprotein Fractions in the Rat
Source: PLoS One. 2014 Oct 8;9(10):e109983. doi: 10.1371/journal.pone.0109983 (PMC4190321; doi:10.1371/journal.pone.0109983)
Supplement: Table S3 — Triacylglycerol concentration in major lipoprotein fractions in the PXO recombinant inbred strain panel and its progenitor strains, BXH2/Cub and SHR- Lx . (PDF) [file pone.0109983.s003.pdf]

	Total triacylglycerol [mg/dL]			CM triacylglycerol [mg/dL]			VLDL triacylglycerol [mg/dL]			LDL triacylglycerol [mg/dL]			HDL triacylglycerol [mg/dL]	
**STRAIN**	**mean**	**SEM**	**STRAIN**	**mean**	**SEM**	**STRAIN**	**mean**	**SEM**	**STRAIN**	**mean**	**SEM**	**STRAIN**	**mean**	**SEM**
**PXO10**	23.5	*2.6*	**PXO6-1**	0.09	*0.03*	**PXO6-1**	5.6	*0.6*	**PXO10**	7.1	*0.4*	**PXO3-1**	2.58	*0.10*
**PXO6-1**	27.0	*1.0*	**PXO6-3**	0.20	*0.05*	**PXO10**	12.9	*2.0*	**PXO3-1**	8.0	*0.3*	**PXO6-1**	2.86	*0.24*
**PXO3-1**	27.4	*0.9*	**PXO6-2**	0.27	*0.09*	**PXO6-3**	13.1	*1.3*	**SHR-Lx**	10.2	*0.5*	**PXO9**	2.87	*0.16*
**BXH2**	30.1	*1.7*	**PXO3-1**	0.29	*0.02*	**BXH2**	15.3	*1.0*	**PXO9**	10.2	*0.9*	**PXO10**	2.97	*0.16*
**PXO9**	31.0	*3.1*	**PXO3-2**	0.36	*0.09*	**PXO6-2**	15.3	*2.8*	**BXH2**	10.7	*1.1*	**BXH2**	3.07	*0.17*
**PXO2**	31.9	*2.2*	**PXO4**	0.37	*0.10*	**PXO2**	16.5	*1.8*	**PXO2**	11.3	*0.5*	**PXO2**	3.20	*0.23*
**PXO6-3**	35.3	*1.2*	**PXO7-1**	0.42	*0.12*	**PXO3-1**	16.5	*1.0*	**PXO1**	12.6	*0.7*	**SHR-Lx**	3.31	*0.14*
**SHR-Lx**	35.6	*3.3*	**SHR-Lx**	0.53	*0.08*	**PXO9**	17.4	*2.2*	**PXO3-2**	14.8	*0.6*	**PXO3-2**	3.68	*0.40*
**PXO3-2**	37.4	*3.8*	**PXO9**	0.54	*0.11*	**PXO3-2**	18.5	*3.1*	**PXO5-1**	16.2	*0.6*	**PXO7-1**	3.71	*0.13*
**PXO6-2**	40.6	*3.9*	**PXO10**	0.54	*0.13*	**SHR-Lx**	21.5	*2.6*	**PXO5-2**	16.6	*0.9*	**PXO6-2**	3.72	*0.13*
**PXO1**	42.6	*3.5*	**PXO8-2**	0.84	*0.11*	**PXO1**	25.0	*2.8*	**PXO8-1**	17.3	*0.9*	**PXO1**	3.78	*0.14*
**PXO8-1**	50.7	*3.8*	**PXO8-1**	0.85	*0.07*	**PXO4**	27.6	*4.5*	**PXO8-2**	17.4	*0.8*	**PXO8-1**	3.85	*0.33*
**PXO7-1**	51.9	*3.6*	**PXO2**	0.98	*0.19*	**PXO7-1**	27.7	*3.1*	**PXO6-3**	17.6	*0.3*	**PXO4**	4.09	*0.18*
**PXO4**	53.6	*4.3*	**BXH2**	1.03	*0.23*	**PXO8-1**	28.6	*2.6*	**PXO6-1**	18.5	*0.5*	**PXO8-2**	4.19	*0.25*
**PXO8-2**	59.4	*4.8*	**PXO1**	1.16	*0.16*	**PXO8-2**	36.9	*4.0*	**PXO7-1**	20.0	*0.8*	**PXO6-3**	4.31	*0.18*
**PXO5-1**	88.8	*6.8*	**PXO5-1**	2.27	*0.40*	**PXO5-1**	64.7	*6.1*	**PXO6-2**	21.3	*1.1*	**PXO5-2**	5.21	*0.16*
**PXO5-2**	92.4	*6.6*	**PXO5-2**	3.85	*0.31*	**PXO5-2**	66.7	*5.8*	**PXO4**	21.6	*1.1*	**PXO5-1**	5.55	*0.48*

Supplementary Table S3. Triacylglycerol concentration in major lipoprotein fractions in the PXO recombinant inbred strain panel and its progenitor strains, BXH2/Cub and SHR-Lx. CM - chylomicron, VLDL - very low-density lipoprotein, LDL - low density lipoprotein, HDL - high-density lipoprotein.
