# Supplementary material for: Genomic Determinants of Triglyceride and Cholesterol Distribution into Lipoprotein Fractions in the Rat
Source: PLoS One. 2014 Oct 8;9(10):e109983. doi: 10.1371/journal.pone.0109983 (PMC4190321; doi:10.1371/journal.pone.0109983)
Supplement: Table S4 — Profile of sizes of major lipoprotein particles in the PXO recombinant inbred strain panel and its progenitor strains, BXH2/Cub and SHR- Lx . (PDF) [file pone.0109983.s004.pdf]

	VLDL size [nm]			LDL size [nm]			HDL size [nm]	
**STRAIN**	**mean**	**SEM**	**STRAIN**	**mean**	**SEM**	**STRAIN**	**mean**	**SEM**
**PXO6-1**	38.6	*0.4*	**PXO3-2**	20.36	*0.05*	**PXO6-1**	12.12	*0.02*
**PXO7-1**	39.2	*0.2*	**PXO3-1**	20.51	*0.12*	**PXO5-1**	12.35	*0.07*
**PXO6-2**	39.2	*0.2*	**SHR-Lx**	20.64	*0.05*	**PXO7-1**	12.35	*0.02*
**PXO4**	39.5	*0.4*	**PXO6-3**	20.77	*0.17*	**PXO6-2**	12.39	*0.02*
**PXO3-2**	39.5	*0.3*	**PXO8-2**	20.81	*0.05*	**PXO6-3**	12.43	*0.06*
**PXO6-3**	39.7	*0.2*	**PXO6-2**	20.90	*0.07*	**PXO5-2**	12.45	*0.04*
**PXO8-1**	40.1	*0.2*	**PXO8-1**	21.07	*0.10*	**PXO8-1**	12.55	*0.03*
**PXO3-1**	40.4	*0.2*	**PXO4**	21.10	*0.10*	**BXH2**	12.57	*0.06*
**SHR-Lx**	40.5	*0.2*	**PXO1**	21.16	*0.21*	**PXO3-2**	12.58	*0.03*
**PXO8-2**	40.8	*0.2*	**PXO2**	21.20	*0.17*	**PXO8-2**	12.65	*0.03*
**PXO5-1**	41.5	*0.3*	**PXO5-1**	21.21	*0.18*	**PXO4**	12.70	*0.02*
**PXO10**	42.1	*0.3*	**PXO7-1**	21.44	*0.04*	**PXO3-1**	12.71	*0.02*
**PXO1**	42.2	*0.3*	**PXO9**	21.46	*0.08*	**PXO10**	12.73	*0.03*
**PXO9**	42.5	*0.2*	**PXO6-1**	21.47	*0.15*	**PXO2**	12.76	*0.03*
**BXH2**	42.6	*0.5*	**PXO10**	21.49	*0.09*	**PXO1**	12.81	*0.02*
**PXO2**	42.9	*0.4*	**BXH2**	21.82	*0.13*	**PXO9**	12.84	*0.05*
**PXO5-2**	43.0	*0.3*	**PXO5-2**	21.96	*0.19*	**SHR-Lx**	13.00	*0.03*

Supplementary Table S4. Profile of sizes of major lipoprotein particles in the PXO recombinant inbred strain panel and its progenitor strains, BXH2/Cub and SHR-Lx. VLDL - very low-density lipoprotein, LDL - low density lipoprotein, HDL - high-density lipoprotein.
