# Supplementary material for: Genomic Determinants of Triglyceride and Cholesterol Distribution into Lipoprotein Fractions in the Rat
Source: PLoS One. 2014 Oct 8;9(10):e109983. doi: 10.1371/journal.pone.0109983 (PMC4190321; doi:10.1371/journal.pone.0109983)
Supplement: Table S5 — Cholesterol concentration in individual lipoprotein fractions l in the PXO recombinant inbred strain panel and its progenitor strains, BXH2/Cub and SHR- Lx . (PDF) [file pone.0109983.s005.pdf]

	Cholesterol fraction 1 [mg/dL]		Cholesterol fraction 2 [mg/dL]		Cholesterol fraction 3 [mg/dL]		Cholesterol fraction 4 [mg/dL]		Cholesterol fraction 5 [mg/dL]		Cholesterol fraction 6 [mg/dL]		Cholesterol fraction 7 [mg/dL]		Cholesterol fraction 8 [mg/dL]		Cholesterol fraction 9 [mg/dL]		Cholesterol fraction 10 [mg/dL]	
	chylomicron		chylomicron		large VLDL		large VLDL		large VLDL		medium VLDL		small VLDL		large LDL		medium LDL		small LDL	
**STRAIN**	**mean**	**SEM**	**mean**	**SEM**	**mean**	**SEM**	**mean**	**SEM**	**mean**	**SEM**	**mean**	**SEM**	**mean**	**SEM**	**mean**	**SEM**	**mean**	**SEM**	**mean**	**SEM**
**BXH2**	2.11E-03	*9.75E-04*	0.16	*0.04*	0.16	*0.03*	0.51	*0.09*	1.37	*0.20*	1.69	*0.24*	1.08	*0.15*	1.58	*0.19*	2.84	*0.25*	3.55	*0.30*
**SHR-Lx**	1.32E-03	*3.73E-04*	0.07	*0.01*	0.10	*0.01*	0.46	*0.05*	1.90	*0.17*	2.71	*0.20*	1.68	*0.11*	1.92	*0.09*	2.34	*0.06*	3.14	*0.07*
**PXO1**	4.44E-04	*4.44E-04*	0.12	*0.02*	0.16	*0.02*	0.67	*0.10*	2.15	*0.34*	2.68	*0.42*	1.66	*0.26*	2.08	*0.32*	3.08	*0.39*	4.17	*0.31*
**PXO2**	7.67E-04	*7.67E-04*	0.17	*0.03*	0.20	*0.03*	0.60	*0.09*	1.34	*0.17*	1.66	*0.17*	1.19	*0.09*	2.12	*0.20*	3.74	*0.33*	4.30	*0.27*
**PXO3-1**	0	*0*	0.02	*0.00*	0.03	*0.01*	0.16	*0.02*	0.67	*0.05*	0.91	*0.06*	0.49	*0.03*	0.57	*0.05*	1.20	*0.08*	2.03	*0.10*
**PXO3-2**	1.17E-04	*1.17E-04*	0.02	*0.00*	0.01	*0.004*	0.10	*0.01*	0.41	*0.06*	0.62	*0.06*	0.38	*0.03*	0.38	*0.02*	0.54	*0.06*	2.14	*0.16*
**PXO4**	4.67E-04	*4.67E-04*	0.03	*0.01*	0.05	*0.01*	0.25	*0.05*	0.91	*0.18*	1.34	*0.15*	0.88	*0.04*	1.02	*0.05*	2.17	*0.25*	4.92	*0.45*
**PXO5-1**	0	*0*	0.16	*0.03*	0.17	*0.02*	0.60	*0.06*	2.04	*0.17*	2.43	*0.19*	1.07	*0.07*	1.02	*0.10*	1.36	*0.23*	2.01	*0.26*
**PXO5-2**	1.22E-03	*8.06E-04*	0.42	*0.05*	0.54	*0.08*	1.53	*0.19*	3.43	*0.28*	3.42	*0.22*	1.76	*0.15*	2.42	*0.25*	3.28	*0.25*	3.21	*0.20*
**PXO6-1**	4.83E-04	*4.83E-04*	0.01	*0.00*	0.01	*0.003*	0.05	*0.005*	0.16	*0.03*	0.30	*0.05*	0.29	*0.04*	0.40	*0.05*	1.04	*0.20*	3.33	*0.31*
**PXO6-2**	6.80E-04	*6.21E-04*	0.02	*0.01*	0.02	*0.005*	0.11	*0.02*	0.38	*0.05*	0.61	*0.08*	0.46	*0.05*	0.54	*0.04*	0.89	*0.10*	3.31	*0.32*
**PXO6-3**	1.18E-03	*4.32E-04*	0.01	*0.00*	0.02	*0.005*	0.11	*0.01*	0.35	*0.04*	0.51	*0.05*	0.36	*0.03*	0.42	*0.03*	0.67	*0.10*	2.51	*0.29*
**PXO7-1**	0	*0*	0.02	*0.01*	0.03	*0.01*	0.17	*0.02*	0.70	*0.08*	1.20	*0.11*	0.82	*0.04*	0.81	*0.03*	1.28	*0.03*	3.20	*0.10*
**PXO8-1**	8.33E-05	*8.33E-05*	0.06	*0.01*	0.05	*0.01*	0.25	*0.03*	0.95	*0.07*	1.39	*0.10*	0.80	*0.05*	0.82	*0.06*	1.40	*0.13*	2.91	*0.17*
**PXO8-2**	0	*0*	0.05	*0.01*	0.06	*0.01*	0.32	*0.03*	1.12	*0.11*	1.43	*0.13*	0.75	*0.05*	0.72	*0.04*	1.07	*0.07*	2.60	*0.08*
**PXO9**	0	*0*	0.08	*0.02*	0.17	*0.02*	0.75	*0.10*	2.09	*0.34*	2.44	*0.40*	1.57	*0.23*	2.31	*0.23*	3.49	*0.19*	3.80	*0.19*
**PXO10**	3.72E-03	*2.24E-03*	0.12	*0.02*	0.20	*0.02*	0.74	*0.06*	2.16	*0.14*	2.82	*0.16*	1.89	*0.09*	2.34	*0.10*	2.85	*0.09*	3.33	*0.08*

	Cholesterol fraction 11 [mg/dL]		Cholesterol fraction 12 [mg/dL]		Cholesterol fraction 13 [mg/dL]		Cholesterol fraction 14 [mg/dL]		Cholesterol fraction 15 [mg/dL]		Cholesterol fraction 16 [mg/dL]		Cholesterol fraction 17 [mg/dL]		Cholesterol fraction 18 [mg/dL]		Cholesterol fraction 19 [mg/dL]		Cholesterol fraction 20 [mg/dL]	
	very small LDL		very small LDL		very small LDL		very large HDL		very large HDL		large HDL		medium HDL		small HDL		very small HDL		very small HDL	
**STRAIN**	**mean**	**SEM**	**mean**	**SEM**	**mean**	**SEM**	**mean**	**SEM**	**mean**	**SEM**	**mean**	**SEM**	**mean**	**SEM**	**mean**	**SEM**	**mean**	**SEM**	**mean**	**SEM**
**BXH2**	2.33	*0.18*	2.29	*0.24*	3.10	*0.30*	5.52	*0.28*	6.62	*0.49*	9.63	*1.25*	3.78	*0.39*	1.29	*0.08*	0.52	*0.02*	0.64	*0.03*
**SHR-Lx**	2.88	*0.12*	5.15	*0.18*	6.13	*0.17*	7.92	*0.16*	6.80	*0.17*	6.73	*0.29*	2.19	*0.12*	0.99	*0.04*	0.43	*0.02*	0.62	*0.01*
**PXO1**	3.32	*0.20*	4.55	*0.09*	5.17	*0.18*	7.11	*0.34*	7.02	*0.47*	8.23	*0.67*	2.92	*0.23*	1.15	*0.07*	0.46	*0.02*	0.66	*0.03*
**PXO2**	2.52	*0.13*	3.53	*0.11*	6.51	*0.32*	11.39	*0.60*	11.40	*0.56*	14.24	*0.72*	5.60	*0.23*	1.89	*0.06*	0.81	*0.03*	0.91	*0.02*
**PXO3-1**	1.39	*0.06*	1.71	*0.09*	3.38	*0.16*	8.11	*0.27*	11.55	*0.31*	14.44	*0.42*	4.33	*0.14*	1.43	*0.04*	0.59	*0.01*	0.74	*0.02*
**PXO3-2**	3.03	*0.15*	2.14	*0.10*	2.48	*0.19*	7.73	*0.45*	12.62	*0.62*	16.88	*0.80*	5.61	*0.29*	1.79	*0.11*	0.75	*0.06*	0.82	*0.04*
**PXO4**	4.31	*0.35*	3.17	*0.10*	3.88	*0.21*	8.43	*0.28*	12.20	*0.26*	15.41	*0.37*	4.70	*0.19*	1.47	*0.04*	0.61	*0.02*	0.79	*0.02*
**PXO5-1**	1.47	*0.19*	1.79	*0.30*	2.79	*0.49*	5.60	*0.64*	7.79	*0.64*	13.66	*0.63*	6.00	*0.27*	1.83	*0.07*	0.72	*0.03*	0.77	*0.03*
**PXO5-2**	1.92	*0.13*	2.74	*0.17*	3.77	*0.29*	6.23	*0.43*	7.10	*0.48*	10.83	*0.71*	4.74	*0.17*	1.58	*0.03*	0.78	*0.04*	0.90	*0.04*
**PXO6-1**	3.51	*0.17*	1.82	*0.09*	0.86	*0.06*	2.50	*0.16*	6.58	*0.31*	13.65	*0.62*	5.54	*0.27*	1.61	*0.07*	0.63	*0.03*	0.64	*0.02*
**PXO6-2**	4.53	*0.25*	2.83	*0.07*	1.86	*0.10*	5.81	*0.35*	11.48	*0.54*	18.66	*0.66*	6.57	*0.24*	2.00	*0.08*	0.79	*0.04*	0.82	*0.03*
**PXO6-3**	3.48	*0.22*	2.29	*0.11*	1.65	*0.16*	5.64	*0.67*	11.44	*0.71*	17.44	*0.50*	5.88	*0.22*	1.74	*0.07*	0.72	*0.04*	0.79	*0.02*
**PXO7-1**	3.24	*0.15*	1.92	*0.10*	1.78	*0.08*	4.57	*0.21*	8.55	*0.36*	15.02	*0.21*	5.56	*0.08*	1.55	*0.03*	0.63	*0.01*	0.69	*0.02*
**PXO8-1**	2.61	*0.10*	1.97	*0.08*	2.85	*0.19*	7.45	*0.44*	11.98	*0.46*	17.52	*0.24*	5.80	*0.13*	1.72	*0.04*	0.69	*0.01*	0.80	*0.01*
**PXO8-2**	2.67	*0.16*	2.01	*0.09*	2.88	*0.16*	8.02	*0.29*	12.59	*0.36*	16.67	*0.63*	5.02	*0.27*	1.57	*0.07*	0.63	*0.03*	0.80	*0.03*
**PXO9**	2.52	*0.16*	3.50	*0.27*	5.25	*0.24*	8.58	*0.43*	8.40	*0.73*	9.72	*1.25*	3.59	*0.45*	1.32	*0.11*	0.58	*0.06*	0.71	*0.04*
**PXO10**	2.84	*0.09*	3.76	*0.22*	4.31	*0.24*	5.89	*0.33*	5.16	*0.33*	6.06	*0.49*	2.65	*0.25*	1.11	*0.08*	0.48	*0.03*	0.65	*0.02*

Supplementary Table S5. Cholesterol concentration in individual lipoprotein fractions l in the PXO recombinant inbred strain panel and its progenitor strains, BXH2/Cub and SHR-Lx. CM - chylomicron, VLDL - very low-density lipoprotein, LDL - low density lipoprotein, HDL - high-density lipoprotein.
