# Supplementary material for: Genomic Determinants of Triglyceride and Cholesterol Distribution into Lipoprotein Fractions in the Rat
Source: PLoS One. 2014 Oct 8;9(10):e109983. doi: 10.1371/journal.pone.0109983 (PMC4190321; doi:10.1371/journal.pone.0109983)
Supplement: Table S6 — Triacylglycerol concentration in individual lipoprotein fractions l in the PXO recombinant inbred strain panel and its progenitor strains, BXH2/Cub and SHR- Lx . (PDF) [file pone.0109983.s006.pdf]

	Triacylglycerol fraction 1 [mg/dL]		Triacylglycerol fraction 2 [mg/dL]		Triacylglycerol fraction 3 [mg/dL]		Triacylglycerol fraction 4 [mg/dL]		Triacylglycerol fraction 5 [mg/dL]		Triacylglycerol fraction 6 [mg/dL]		Triacylglycerol fraction 7 [mg/dL]		Triacylglycerol fraction 8 [mg/dL]		Triacylglycerol fraction 9 [mg/dL]		Triacylglycerol fraction 10 [mg/dL]	
	chylomicron		chylomicron		large VLDL		large VLDL		large VLDL		medium VLDL		small VLDL		large LDL		medium LDL		small LDL	
STRAIN	mean	SEM	mean	SEM	mean	SEM	mean	SEM	mean	SEM	mean	SEM	mean	SEM	mean	SEM	mean	SEM	mean	SEM
BXH2	1.18E-02	*6.86E-03*	1.02	*0.23*	0.89	*0.15*	2.64	*0.34*	5.19	*0.40*	4.40	*0.29*	2.13	*0.18*	2.10	*0.17*	2.71	*0.28*	2.97	*0.40*
SHR-Lx	2.45E-02	*1.03E-02*	0.51	*0.08*	0.56	*0.08*	2.42	*0.35*	7.07	*0.95*	7.75	*0.90*	3.72	*0.36*	3.01	*0.24*	2.37	*0.13*	2.10	*0.07*
PXO1	1.37E-02	*6.67E-03*	1.15	*0.16*	1.06	*0.14*	3.95	*0.48*	9.07	*1.14*	7.70	*0.90*	3.25	*0.33*	2.77	*0.24*	2.81	*0.18*	3.17	*0.16*
PXO2	2.38E-02	*7.52E-03*	0.95	*0.19*	1.06	*0.19*	3.18	*0.48*	5.48	*0.65*	4.47	*0.39*	2.30	*0.13*	2.43	*0.11*	2.92	*0.19*	2.91	*0.15*
PXO3-1	1.64E-02	*5.59E-03*	0.27	*0.02*	0.35	*0.04*	1.72	*0.17*	5.69	*0.41*	6.21	*0.32*	2.59	*0.13*	1.93	*0.08*	1.81	*0.07*	2.10	*0.15*
PXO3-2	6.23E-03	*5.01E-03*	0.35	*0.08*	0.32	*0.06*	1.58	*0.36*	5.77	*1.19*	7.34	*1.19*	3.52	*0.41*	2.62	*0.25*	1.97	*0.14*	3.47	*0.16*
PXO4	2.70E-02	*1.77E-02*	0.34	*0.10*	0.52	*0.15*	2.65	*0.67*	8.53	*2.05*	10.51	*1.43*	5.40	*0.31*	4.45	*0.17*	4.15	*0.30*	5.64	*0.48*
PXO5-1	2.75E-03	*2.22E-03*	2.27	*0.40*	2.31	*0.30*	8.20	*0.68*	22.85	*2.58*	22.73	*2.50*	8.66	*0.76*	5.71	*0.36*	3.58	*0.13*	3.01	*0.15*
PXO5-2	1.60E-02	*8.55E-03*	3.84	*0.32*	3.85	*0.33*	11.63	*1.15*	24.43	*2.54*	19.47	*1.70*	7.36	*0.59*	5.62	*0.41*	4.11	*0.25*	3.10	*0.15*
PXO6-1	1.45E-02	*6.53E-03*	0.07	*0.03*	0.10	*0.03*	0.40	*0.05*	1.32	*0.19*	2.15	*0.29*	1.61	*0.15*	1.64	*0.12*	2.37	*0.28*	5.67	*0.30*
PXO6-2	5.28E-03	*2.96E-03*	0.26	*0.09*	0.25	*0.10*	1.42	*0.32*	4.23	*0.80*	5.82	*1.03*	3.57	*0.61*	3.10	*0.48*	2.71	*0.26*	5.16	*0.32*
PXO6-3	2.86E-02	*2.34E-02*	0.17	*0.04*	0.27	*0.04*	1.31	*0.15*	3.80	*0.42*	4.87	*0.52*	2.88	*0.26*	2.52	*0.21*	2.25	*0.07*	4.26	*0.32*
PXO7-1	8.90E-03	*3.82E-03*	0.41	*0.12*	0.47	*0.09*	2.30	*0.33*	7.72	*1.03*	11.03	*1.17*	6.18	*0.50*	4.89	*0.34*	3.90	*0.16*	4.83	*0.15*
PXO8-1	1.16E-02	*3.89E-03*	0.84	*0.07*	0.70	*0.08*	2.85	*0.33*	8.90	*0.83*	10.91	*0.99*	5.29	*0.44*	4.06	*0.30*	3.36	*0.19*	4.27	*0.19*
PXO8-2	1.56E-02	*5.69E-03*	0.82	*0.10*	0.90	*0.12*	4.52	*0.62*	12.67	*1.55*	13.13	*1.33*	5.73	*0.45*	4.23	*0.29*	3.15	*0.15*	4.05	*0.18*
PXO9	1.19E-02	*2.61E-03*	0.52	*0.11*	0.80	*0.13*	3.15	*0.44*	6.21	*0.79*	4.95	*0.62*	2.27	*0.26*	2.33	*0.22*	2.73	*0.27*	2.59	*0.27*
PXO10	7.07E-03	*7.07E-03*	0.53	*0.12*	0.64	*0.14*	2.12	*0.41*	4.54	*0.75*	3.85	*0.53*	1.76	*0.20*	1.66	*0.14*	1.62	*0.09*	1.67	*0.08*

	Triacylglycerol fraction 11 [mg/dL]		Triacylglycerol fraction 12 [mg/dL]		Triacylglycerol fraction 13 [mg/dL]		Triacylglycerol fraction 14 [mg/dL]		Triacylglycerol fraction 15 [mg/dL]		Triacylglycerol fraction 16 [mg/dL]		Triacylglycerol fraction 17 [mg/dL]		Triacylglycerol fraction 18 [mg/dL]		Triacylglycerol fraction 19 [mg/dL]		Triacylglycerol fraction 20 [mg/dL]	
	very small LDL		very small LDL		very small LDL		very large HDL		very large HDL		large HDL		medium HDL		small HDL		very small HDL		very small HDL	
STRAIN	mean	SEM	mean	SEM	mean	SEM	mean	SEM	mean	SEM	mean	SEM	mean	SEM	mean	SEM	mean	SEM	mean	SEM
BXH2	2.33	*0.24*	2.29	*0.06*	3.10	*0.01*	5.52	*0.02*	6.62	*0.03*	9.63	*0.05*	3.78	*0.03*	1.29	*0.03*	0.52	*0.02*	0.64	*0.02*
SHR-Lx	2.88	*0.04*	5.15	*0.04*	6.13	*0.03*	7.92	*0.03*	6.80	*0.03*	6.73	*0.04*	2.19	*0.02*	0.99	*0.02*	0.43	*0.02*	0.62	*0.02*
PXO1	3.32	*0.12*	4.55	*0.06*	5.17	*0.04*	7.11	*0.04*	7.02	*0.04*	8.23	*0.04*	2.92	*0.02*	1.15	*0.01*	0.46	*0.01*	0.66	*0.02*
PXO2	2.52	*0.06*	3.53	*0.05*	6.51	*0.03*	11.39	*0.04*	11.40	*0.04*	14.24	*0.05*	5.60	*0.03*	1.89	*0.02*	0.81	*0.02*	0.91	*0.04*
PXO3-1	1.39	*0.10*	1.71	*0.05*	3.38	*0.01*	8.11	*0.01*	11.55	*0.01*	14.44	*0.02*	4.33	*0.04*	1.43	*0.05*	0.59	*0.03*	0.74	*0.03*
PXO3-2	3.03	*0.08*	2.14	*0.06*	2.48	*0.02*	7.73	*0.01*	12.62	*0.02*	16.88	*0.04*	5.61	*0.10*	1.79	*0.14*	0.75	*0.09*	0.82	*0.04*
PXO4	4.31	*0.31*	3.17	*0.10*	3.88	*0.03*	8.43	*0.04*	12.20	*0.05*	15.41	*0.06*	4.70	*0.02*	1.47	*0.03*	0.61	*0.02*	0.79	*0.02*
PXO5-1	1.47	*0.13*	1.79	*0.05*	2.79	*0.01*	5.60	*0.02*	7.79	*0.05*	13.66	*0.11*	6.00	*0.11*	1.83	*0.11*	0.72	*0.07*	0.77	*0.06*
PXO5-2	1.92	*0.08*	2.74	*0.03*	3.77	*0.03*	6.23	*0.04*	7.10	*0.02*	10.83	*0.02*	4.74	*0.02*	1.58	*0.03*	0.78	*0.03*	0.90	*0.06*
PXO6-1	3.51	*0.22*	1.82	*0.19*	0.86	*0.04*	2.50	*0.03*	6.58	*0.03*	13.65	*0.04*	5.54	*0.03*	1.61	*0.04*	0.63	*0.03*	0.64	*0.07*
PXO6-2	4.53	*0.33*	2.83	*0.10*	1.86	*0.02*	5.81	*0.02*	11.48	*0.05*	18.66	*0.10*	6.57	*0.06*	2.00	*0.02*	0.79	*0.04*	0.82	*0.03*
PXO6-3	3.48	*0.16*	2.29	*0.06*	1.65	*0.03*	5.64	*0.04*	11.44	*0.04*	17.44	*0.04*	5.88	*0.05*	1.74	*0.07*	0.72	*0.06*	0.79	*0.03*
PXO7-1	3.24	*0.19*	1.92	*0.10*	1.78	*0.03*	4.57	*0.03*	8.55	*0.03*	15.02	*0.05*	5.56	*0.02*	1.55	*0.03*	0.63	*0.02*	0.69	*0.04*
PXO8-1	2.61	*0.18*	1.97	*0.11*	2.85	*0.04*	7.45	*0.05*	11.98	*0.07*	17.52	*0.07*	5.80	*0.05*	1.72	*0.06*	0.69	*0.03*	0.80	*0.03*
PXO8-2	2.67	*0.22*	2.01	*0.14*	2.88	*0.04*	8.02	*0.04*	12.59	*0.05*	16.67	*0.07*	5.02	*0.03*	1.57	*0.02*	0.63	*0.02*	0.80	*0.03*
PXO9	2.52	*0.13*	3.50	*0.05*	5.25	*0.03*	8.58	*0.04*	8.40	*0.05*	9.72	*0.06*	3.59	*0.02*	1.32	*0.02*	0.58	*0.01*	0.71	*0.03*
PXO10	2.84	*0.07*	3.76	*0.06*	4.31	*0.04*	5.89	*0.04*	5.16	*0.04*	6.06	*0.03*	2.65	*0.03*	1.11	*0.04*	0.48	*0.03*	0.65	*0.02*

Supplementary Table S6. Triacylglycerol concentration in individual lipoprotein fractions l in the PXO recombinant inbred strain panel and its progenitor strains, BXH2/Cub and SHR-Lx. CM - chylomicron, VLDL - very low-density lipoprotein, LDL - low density lipoprotein, HDL - high-density lipoprotein.
